# Testing an inverse modeling approach with gradient boosting regression for stroke volume estimation using patient thermodilution data

**DOI:** 10.3389/frai.2025.1530453

**Published:** 2025-03-18

**Authors:** Vasiliki (Vicky) Bikia, Dionysios Adamopoulos, Marco Roffi, Georgios Rovas, Stéphane Noble, François Mach, Nikolaos Stergiopulos

**Affiliations:** ^1^Laboratory of Hemodynamics and Cardiovascular Technology, Institute of Bioengineering, Swiss Federal Institute of Technology, Lausanne, Switzerland; ^2^Department of Internal Medicine, Division of Cardiology, Hôpitaux Universitaires de Genève, Geneva, Switzerland; ^3^Department of Diagnostics, Division of Nuclear Medicine, Hôpitaux Universitaires de Genève, Geneva, Switzerland; ^4^Faculty of Medicine, Department of Medicine, Geneva University, Geneva, Switzerland

**Keywords:** cardiac output, non-invasive monitoring, hemodynamics, blood pressure, supervised learning, gradient boosting

## Abstract

Stroke volume (SV) is a major indicator of cardiovascular function, providing essential information about heart performance and blood flow adequacy. Accurate SV measurement is particularly important for assessing patients with heart failure, managing patients undergoing major surgeries, and delivering optimal care in critical settings. Traditional methods for estimating SV, such as thermodilution, are invasive and unsuitable for routine diagnostics. Non-invasive techniques, although safer and more accessible, often lack the precision and user-friendliness needed for continuous bedside monitoring. We developed a modified method for SV estimation that combines a validated 1-D model of the systemic circulation with machine learning. Our approach replaces the traditional optimization process developed in our previous work, with a regression method, utilizing an in silico-generated dataset of various hemodynamic profiles to create a gradient boosting regression-enabled SV estimator. This dataset accurately mimics the dynamic characteristics of the 1-D model, allowing for precise SV predictions without resource-intensive parameter adjustments. We evaluated our method against SV values derived from the gold standard thermodilution method in 24 patients. The results demonstrated that our approach provides a satisfactory agreement between the predicted and reference data, with a MAE of 16 mL, a normalized RMSE of 21%, a bias of −9.2 mL, and limits of agreement (LoA) of [−47, 28] mL. A correlation coefficient of *r* = 0.7 (*p* < 0.05) was reported, with the predicted SV slightly underestimated (68 ± 23 mL) in comparison to the reference SV (77 ± 26 mL). The significant reduction in computational time of our method for SV assessment should make it suitable for real-time clinical applications.

## 1 Introduction

Monitoring stroke volume (SV) is critical in clinical practice as it provides essential information about the heart’s performance and blood flow adequacy. Accurate SV measurement enables healthcare professionals to assess cardiac function efficiency, identify circulation abnormalities, and guide treatment decisions. This is particularly important for the management of patients with cardiac diseases, heart failure, or those undergoing major surgeries or critical care (Berkenstadt et al., 2001; McKendry et al., 2004; Lees et al., 2009), in order to optimize interventions and prevent complications.

Monitoring biomarkers for vascular and cardiac function plays a crucial role in identifying, treating, and evaluating cardiovascular diseases (Vincent et al., 2015). SV, a major parameter of cardiovascular function, reflects the interdependent performance of the heart and major blood vessels. However, the hemodynamic management of patients using SV remains limited, with patient care primarily guided by simple brachial cuff blood pressure (BP) observations alone (Phillips et al., 2017). Such approaches compromise the usefulness and effectiveness of hemodynamically-guided interventions (Thiel et al., 2009; Meng and Heerdt, 2016).

Thermodilution is considered one of the most reliable and accurate clinical techniques for estimating cardiac output (CO), positioned between the direct Fick method and the indirect Fick method. Stroke volume (SV) is then calculated by dividing CO by heart rate (HR). However, thermodilution is highly invasive and carries a risk of complications, making it unsuitable for routine use. To overcome these limitations, several less but still invasive methods for assessing CO and SV have been developed (Cholley and Singer, 2003; Udy et al., 2012; Jansen et al., 2001), including pulse contour analysis or esophageal Doppler. Yet, their relative invasiveness precludes their use for routine clinical examinations. Alternatively, non-invasive techniques such as inert gas rebreathing, doppler ultrasound, or magnetic resonance imaging (MRI) have been used (Phillips et al., 2017; Guzzetti et al., 2021; Middlemiss et al., 2019). While these methods are non-invasive and reasonably accurate, they are expensive, and require costly equipment and expert staff (Porter et al., 2015). Moreover, none of these methods can be applied for continuous bedside monitoring of SV.

**Figure 1:**
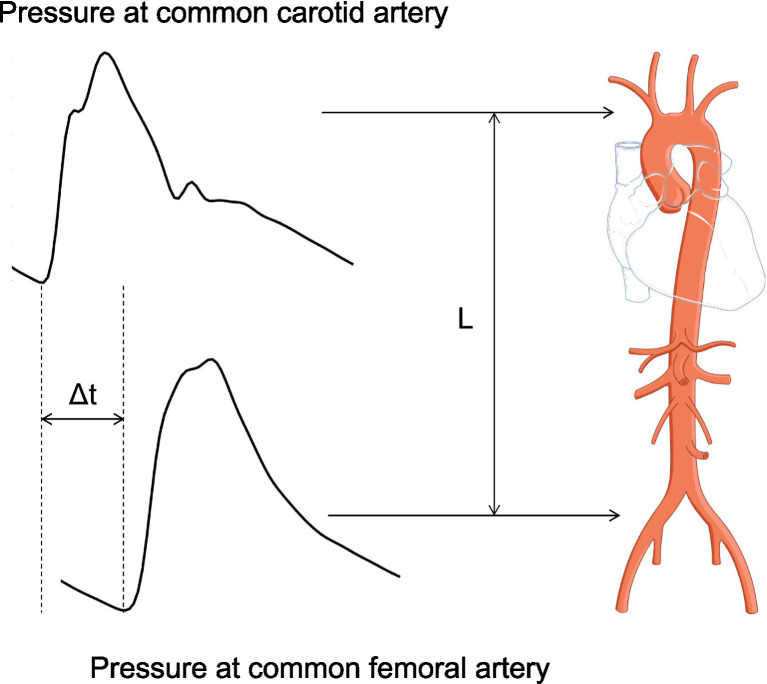
Estimation of cfPWV estimation. The calculation of cfPWV involves two key components: the estimation of *L*, the distance between these two arterial sites, and the measurement of the pulse transit time (Δ*t*). The cfPWV is then calculated using the formula cfPWV = *L*/Δ*t*.

In our prior research, we utilized a one-dimensional (1-D) model of the systemic circulation (Reymond et al., 2009), adjusting its parameters iteratively through multiple runs to derive a personalized profile of an individual’s arterial hemodynamics, and thus estimate SV. Specifically, we applied an inverse-problem solving method to obtain non-invasive estimates of mean aortic flow using age, weight, height, and measurements of brachial BP and carotid-femoral pulse wave velocity (cfPWV) (Bikia et al., 2020). CfPWV can be routinely measured in clinical practice, by calculating the speed at which the blood pressure pulse travels from the carotid artery in the neck to the femoral artery in the thigh. The calculation of cfPWV involves two key components: the estimation of *L*, the distance between these two arterial sites, and the measurement of the pulse transit time (Δ*t*). The approximation of *L* is typically based on the surface distance between the two sites, adjusted to more closely match the path that the pulse wave follows through the body’s arterial network. Often, this involves subtracting a fixed distance from the direct path to better approximate the actual arterial distance, enhancing the accuracy of the cfPWV calculation. The pulse transit time, Δ*t*, is measured as the time difference between the arrival of the pulse wave at the carotid artery and its arrival at the femoral artery, typically using sensors placed at both sites. The cfPWV is then calculated using the formula cfPWV = *L*/Δ*t* (Figure 1). CfPWV exhibits satisfactory repeatability, and has been identified as an independent predictor of clinical outcomes (Laurent et al., 2006), making it a valuable addition to routine BP measurements in risk assessments. Despite the promising results we received while comparing the predictions to reference data in our previous works (Bikia et al., 2020; Bikia et al., 2022), this approach proved to be time-consuming, limiting its potential for real-time monitoring applications.

Machine learning has created tremendous opportunities in the field of health monitoring and prognostics by providing higher quality data insights, due to advanced models and low inference time (Bikia et al., 2021; Wang et al., 2022; Panch et al., 2018). Building upon this foundation, we introduce a modified version of our original method, by replacing the optimization process for tuning the 1-D arterial model with a regression method combined with an in silico-generated dataset of different hemodynamic profiles, creating a gradient boosting regressor-enabled SV estimator. This dataset faithfully mimics the dynamic characteristics and analytical mathematical equations inherent to the model. In addition, in this study, we designed a clinical protocol and compared the predicted values of this new method to SV values derived using the gold standard invasive thermodilution method. By leveraging the in silico-generated data, we sidestep the need for resource-intensive parameter adjustments in real-time scenarios. Instead, the generated content serves as a basis for making predictions on actual human data. This innovative methodology not only streamlines the computational process but also enhances the applicability of our approach, paving the way for more efficient and practical implementation in real-world monitoring applications.

## 2 Materials and methods

### 2.1 Stroke volume estimation approach

**Figure 2:**
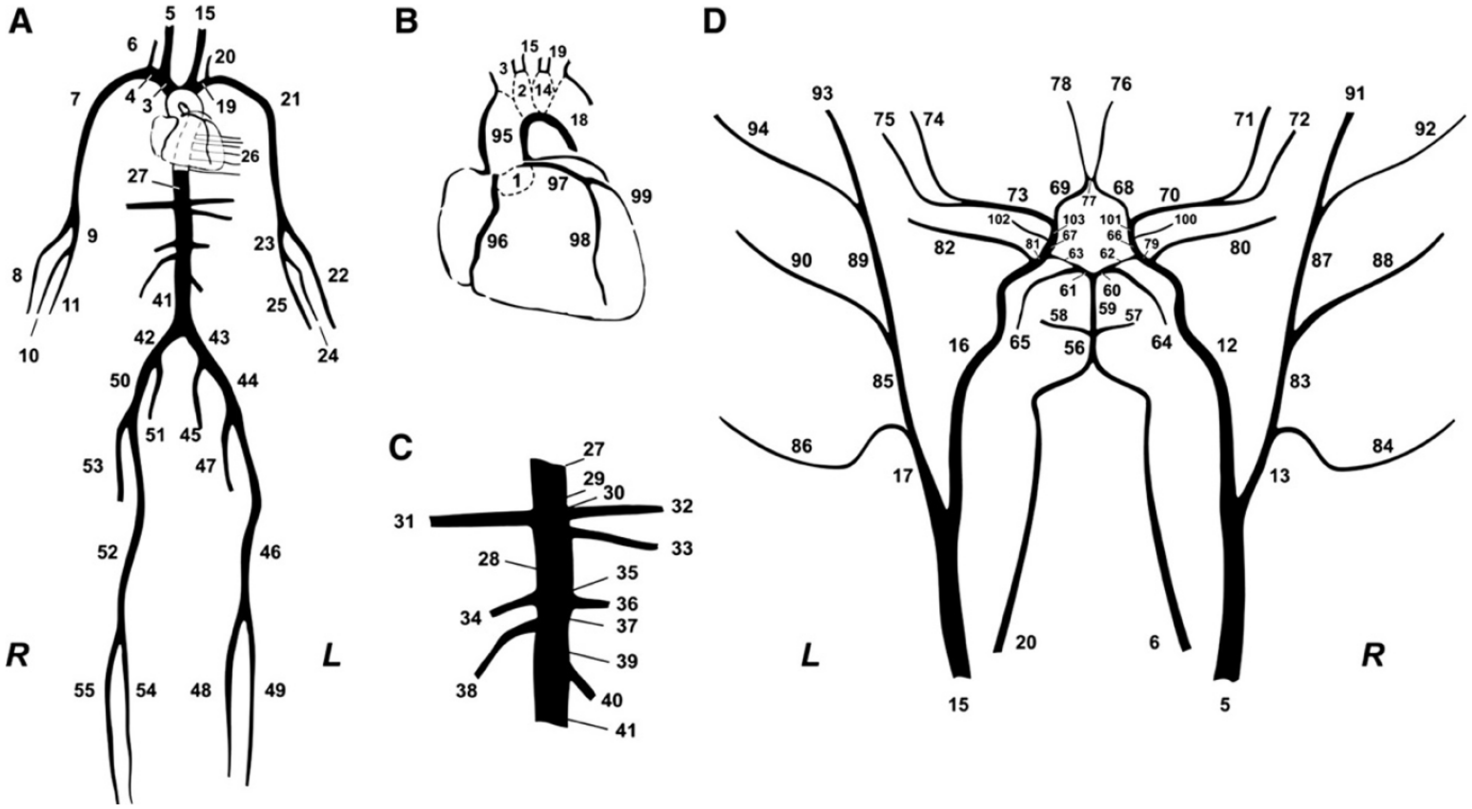
Schematic representation of the model of systemic circulation developed by Reymond et al. (2009). **(A)** Main systemic arterial tree. **(B)** Detail of the aortic arch and the coronary network. **(C)** Detail of the principal abdominal aorta branches. **(D)** Blown-up schematic of the detailed cerebral arterial tree, which is connected via the carotids (segments 5 and 15) and the vertebrals (segments 6 and 20) to the main arterial tree shown in panel **(A)**.

**Figure 3:**
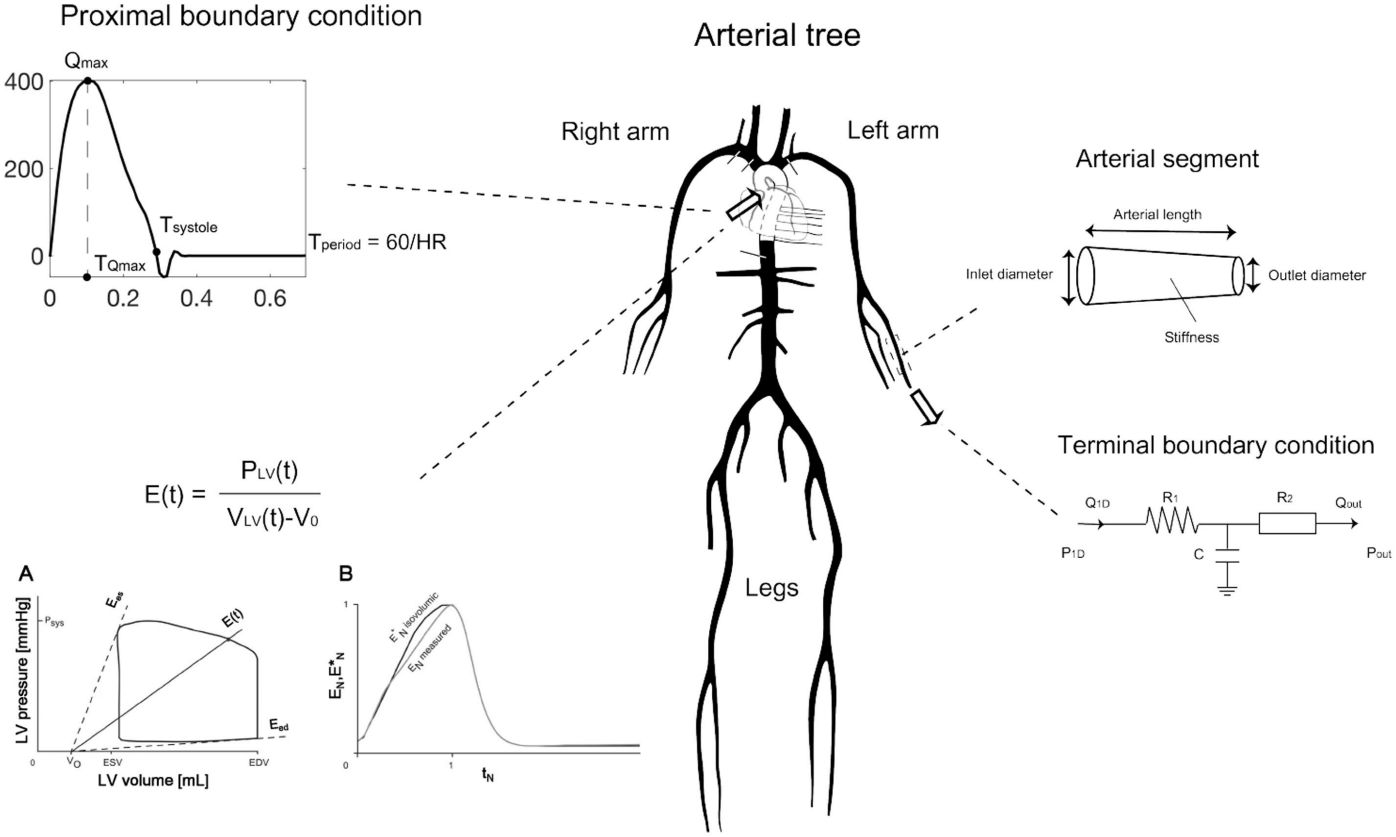
Main mathematical components describing the numerical modeling approach of the 1-D arterial tree computer solver. Adapted from https://infoscience.epfl.ch/entities/publication/79637d9b-89a0-4fc0-8649-50e00810a0b6, with permission.

**Figure 4:**
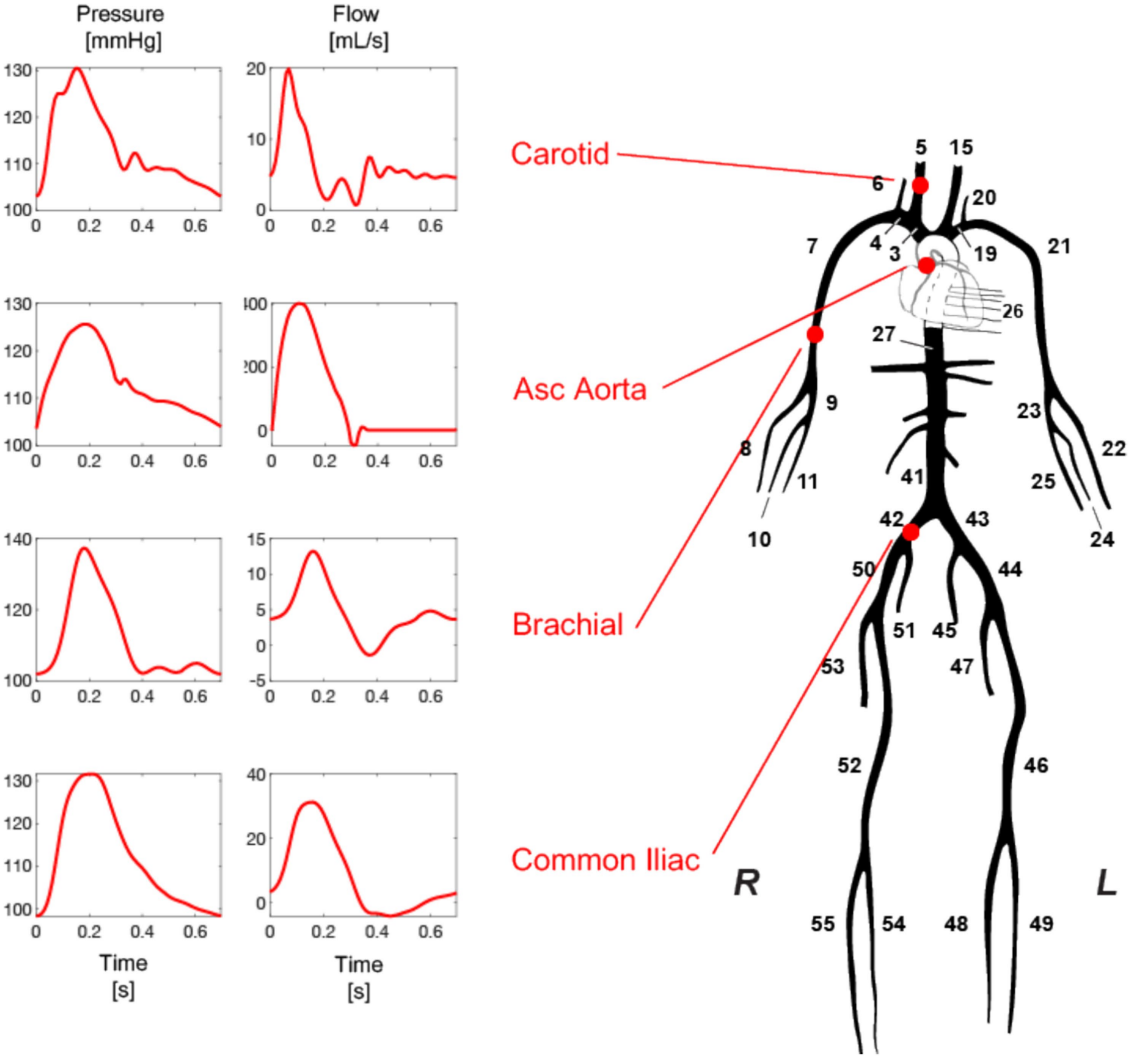
Pressure and flow waveforms are generated at every arterial side of the arterial tree model with every simulation. Adapted from https://infoscience.epfl.ch/entities/publication/79637d9b-89a0-4fc0-8649-50e00810a0b6, with permission.

The method developed in this research builds upon the original inverse problem-solving approach introduced and tested in previous reports (Bikia et al., 2020; Bikia et al., 2022). The core component of the methods involves a comprehensive numerical model of the arterial tree that encompasses all major vessels within the systemic circulation, including the cerebral and coronary circulations (Figure 2). The foundational equations of this model are derived by integrating the longitudinal momentum and continuity equations (Navier–Stokes) across the arterial cross-section. The model solves these governing equations using appropriate boundary conditions (Figure 3), thereby enabling the calculation of flow and pressure across every location within the arterial network (Figure 4). Each arterial segment is represented as a long, tapered tube with compliance characterized as a nonlinear function of pressure and location (Langewouters, 1984) (Figure 3). Terminal vessels are integrated with three-element Windkessel models (Westerhof et al., 2009) (Figure 3), and intimal shear stress is modeled based on the Witzig-Womersley theory proposed (Womersley, 1957). At the proximal end, specifically at the root of the aorta, the model is initialized with an aortic blood flow wave as a proximal boundary condition (Figure 3). Table 1 in the original publications provides a summary of all inputs and outputs of the 1-D cardiovascular model. A detailed description of the 1-D simulator can be found in the original publications (Reymond et al., 2009; Reymond et al., 2011). The model has been used in the past in our own prior work as well as by external researchers, proving its reliability and utility across a wide range of applications (Bikia et al., 2021; Pagoulatou et al., 2019; Reymond et al., 2013; Obeid et al., 2022).

**Table 1:** List of inputs and outputs of the 1-D cardiovascular model.

	Corresponding variable	Value
Inputs		
Cardiac output (L/min)	*CO*	5.5
Heart rate (bpm)	*HR*	75
Ejection time (s)	*ET*	0.23
Arterial distensibility (10^−3^/mmHg)	*C*	(no segments) × 1 vector
Terminal compliances (mL/mmHg)	*Ct*	(no terminal segments) × 1 vector
Peripheral resistances (mmHg·s/mL)	*Rt*	(no terminal segments) × 1 vector
Arterial inlet diameter (cm)	*Din*	(no segments) × 1 vector
Arterial outlet diameter (cm)	*Dout*	(no segments) × 1 vector
Arterial length (cm)	*Len*	(no segments) × 1 vector
Blood density (kg/m^3^)	*ρ*	1,050
Blood viscosity (Pa·s)	*μ*	0.004 s
Outputs		
Pressure waves (mmHg)	*pressures*	(no segments) × (no time points) vector
Flow waves (mL/s)	*flows*	(no segments) × (no time points) vector

This foundational method, as described before (Bikia et al., 2020; Bikia et al., 2022), provides a non-invasive estimation of mean arterial blood flow using peripheral cuff-pressure measurements and cfPWV. The adjustment of a previously validated 1-D arterial tree model (Figure 2) is accomplished through an optimization process. Within this optimization loop, the compliance and resistance of the generic arterial tree model, as well as aortic flow, are iteratively adjusted to ensure that the simulated brachial systolic and diastolic pressures and cfPWV converge with the measured values. This iterative process continues until full convergence is achieved for both brachial pressures and cfPWV.

**Figure 5:**
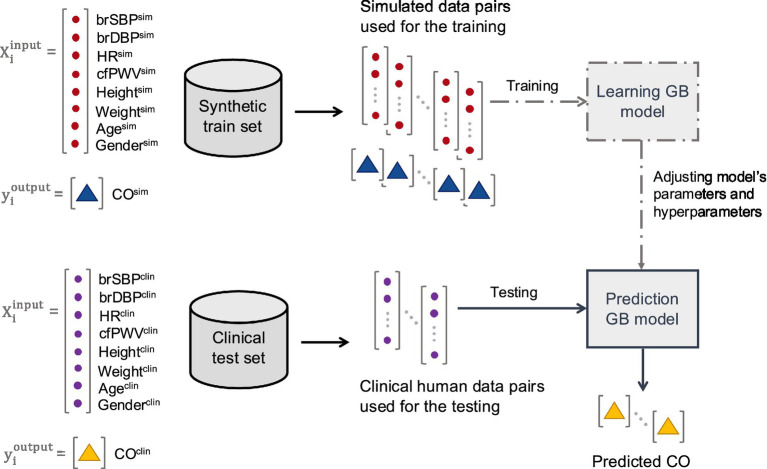
Schematic representation of the training (including hyperparameter tuning) and testing methodological approach.

Given the computational expense of achieving convergence, we sought to enhance efficiency by leveraging a machine learning regression framework. Specifically, we replaced the original model with a gradient boosting regression method trained on a diverse dataset of hemodynamic and demographic profiles generated using the same 1-D arterial tree model (Reymond et al., 2009). The method was then evaluated using data from a patient cohort recruited at the Geneva University Hospitals (HUG). Figure 5 illustrates the methodological approach employed for this study.

#### 2.1.1 Generation of synthetic hemodynamics states

In silico data were generated to simulate various hemodynamic states of the 1-D numerical arterial tree simulation model. The simulation model ran using different combinations of physiologically relevant input model parameters. The distributions of the input model parameters were based on literature data. Given that the literature data are only provided in terms of mean and standard deviation or/and minimum and maximum values, the exact distribution of each parameter was unknown. In addition, varying the parameters while accounting for dependencies between parameters was not feasible due to the lack of sufficient data to inform inter-dependencies. Therefore, the sampling was selected to be random Gaussian. Arterial distensibility, peripheral resistances, and terminal compliances were altered to cover the selected parameter value ranges. Furthermore, the geometry of the arterial network (namely length, inlet diameter, and outlet diameter of the arterial segments) was modified to simulate different body types by adapting the length and the diameter of all arterial vessels. Age and gender were incorporated as factors to vary the model parameters. The literature data drawn from the literature are summarized in Table 2.

**Table 2:** Variation of the input parameters of the 1-D numerical model for the synthetic cases generation.

Model parameter	Gender	Age groups					
		20–29 yrs	30–39 yrs	40–49 yrs	50–59 yrs	60–69 yrs	>70 yrs
Height (cm)	M	175 ± 15					
	F	175 ± 15					
Weight (kg) (Rietzschel et al., 2007)	M	77 ± 10	79 ± 10	86 ± 9	77 ± 9	79 ± 5	75 ± 9
	F	61 ± 7	66 ± 8	65 ± 11	62 ± 6	69 ± 15	62 ± 9
Heart rate (bpm) (McEniery et al., 2005)	M	64 ± 11	63 ± 9	66 ± 11	63 ± 13	65 ± 13	72 ± 13
	F	69 ± 13	59 ± 8	67 ± 12	67 ± 9	67 ± 8	67 ± 13
Aortic distensibility (10^−3^/mmHg) (Chen et al., 1998; Pak et al., 1998; Feldman et al., 1996)	M	7.8 ± 2.5	5.3 ± 1.8	3.8 ± 1.3	3.3 ± 1.6	1.8 ± 1.3	1.3 ± 0.9
	F	8.9 ± 2.5	5.9 ± 2.6	4 ± 1.6	3.1 ± 1.8	1.2 ± 0.8	1.1 ± 0.8
Total peripheral resistance (mmHg·s/mL) (McEniery et al., 2005)	M	1.15 ± 0.26	1.2 ± 0.27	1.28 ± 0.29	1.34 ± 0.31	1.41 ± 0.33	1.49 ± 0.34
	F	1.15 ± 0.26	1.2 ± 0.27	1.28 ± 0.29	1.34 ± 0.31	1.41 ± 0.33	1.49 ± 0.34
Mean arterial pressure (mmHg) (McEniery et al., 2005)	M	89 ± 8	92 ± 8	95 ± 7	95 ± 7	94 ± 7	93 ± 7
	F	86 ± 8	88 ± 9	90 ± 9	93 ± 8	93 ± 8	92 ± 8

##### 2.1.1.1 Proximal boundary condition

Concerning the proximal boundary condition, we engaged in the following systematic logical computations: Given a specific combination of mean arterial pressure (MAP), total peripheral resistance (TPR), and HR, we derive an input value for CO or SV, calculated as CO = MAP/TPR or SV = CO/HR. The adjustment of left ventricular ejection time (ET) is accomplished through the application of the Weissler equation (Weissler et al., 1968) to the provided set of SV and HR values: ET = 0.266 + 0.0011*(SV − 82) − 0.0009*(HR − 73).

##### 2.1.1.2 Anatomical model parameter

The length of each vessel (*Len*) was adjusted to align with the input height in each generated case. The reference state of the arterial tree model is standardized for an individual with a height of 180 cm. We postulated a uniform alteration across all arteries by applying the same scaling factor through multiplication. For instance, when the input height is 170 cm, the scaling factor becomes 170 cm/180 cm = 0.94. Consequently, the length of all arteries is uniformly scaled by a factor of 0.94.

The adjustment of the ascending aortic diameter (*Din*, *Dout*) was informed by data already presented in Wolak et al. (2008), which delineated the aortic diameter as a function of age, gender, and body mass index (BSA). In a concerted manner, all arterial diameters underwent uniform adjustments derived from the variation in aortic diameter. This was achieved through a consistent multiplication process, applying the same scaling factor across all arteries.

##### 2.1.1.3 Arterial distensibility, terminal compliance and resistance

The adjustment of arterial distensibility (*C*), terminal compliance (*Ct*), and terminal resistance (*Rt*) was performed uniformly. Essentially, considering the variation in aortic distensibility as outlined in Table 2, the distensibility and compliance of all arteries were systematically modified to align with the changes in aortic size. Similarly, the resistance of terminal arteries underwent uniform modification, taking into account the distribution of total peripheral resistance (TPR) as detailed in Table 2.

We generated 833 hemodynamics states for 12 age and gender groups, namely $AG_{20-29}^{M}$, $AG_{20-29}^{F}$, $AG_{30-39}^{M}$, $AG_{30-39}^{F}$, $AG_{40-49}^{M}$, $AG_{40-49}^{F}$, $AG_{50-59}^{M}$, $AG_{50-59}^{F}$, $AG_{60-69}^{M}$, $AG_{60-69}^{F}$, $AG_{>70}^{M}$: 10, $AG_{>70}^{F}$. These states simulate the solution space of the 1-D model and mimic the deterministic nature of the solver. Thus, a dataset of 9,996 cases was yielded. From the simulated output blood pressure and flow waveforms, we extracted the investigated parameters for our analysis. BP parameters, such as systolic BP (SBP), and diastolic BP (DBP) were extracted from the simulated waveforms at the left side of the arterial tree.

#### 2.1.2 Gradient boosting regression method

A gradient boosting (GB) algorithm was trained and validated using the in silico-generated data. Hyperparameter tuning was conducted using GridSearch() to find the optimal values for *‘n_estimators’* (60, 80, 100, 120), *‘learning_rate’* (0.2, 0.4, 0.6), ‘max_depth’ (3, 5, 8, 10), *‘max_features’* (‘sqrt’), and *‘min_samples_leaf’* (1, 3, 5). We assumed a 5-fold cross validation scheme and scoring method the *R*^2^ for the hyperparameter tuning. The input vector consisted of age, gender, weight, height, SBP, DBP, PP, MAP, and cfPWV (independent variables) and the output was set to SV (dependent variable). The predicted SV values (SV_pred_) were then compared to the reference SV data obtained from thermodilution (SV_thermo_). No further fine-tuning was performed using the patient data to maintain the integrity of the model evaluation and ensure that the GB regressor remained blind to the *in vivo* data content. The models were developed in Python (Python Software Foundation, Python Language Reference, version 3.10.9)^1^, utilizing the following libraries: pandas (McKinney, 2010), numpy (Harris et al., 2020), matplotlib (Hunter, 2007), scikit-learn (Pedregosa et al., 2011), and scipy (Virtanen et al., 2020).

### 2.2 Patient recruitment and data collection

The data collection for this study was conducted at the HUG and was approved by the Local Ethics Committee of Geneva. The study involved the recruitment of 37 participants selected according to the inclusion and exclusion criteria listed in Supplementary Table S1. Informed consent was obtained from all participants prior to their inclusion in the study. The data consists of non-invasive hemodynamical measurements including conventional sphygmomanometry, applanation tonometry, as well as invasive assessment of SV by the thermodilution method. Given that the invasive SV measurement involves heart catheterization, the study included only patients with a clinical indication for left and/or right heart catheterization as part of their standard diagnostic work up.

#### 2.2.1 Recruitment, screening, and informed consent procedure

A designated healthcare worker identified potential patient participants and contacted them to ask for their participation in the study. The healthcare worker provided detailed information about the study, including its purpose, procedures, expected duration, benefits, and potential discomforts. Upon a positive response, participants were registered in the study and sent an information sheet and consent form via email or post. The formal consent was obtained before the catheterization procedures.

#### 2.2.2 Study procedures

**Figure 6:**
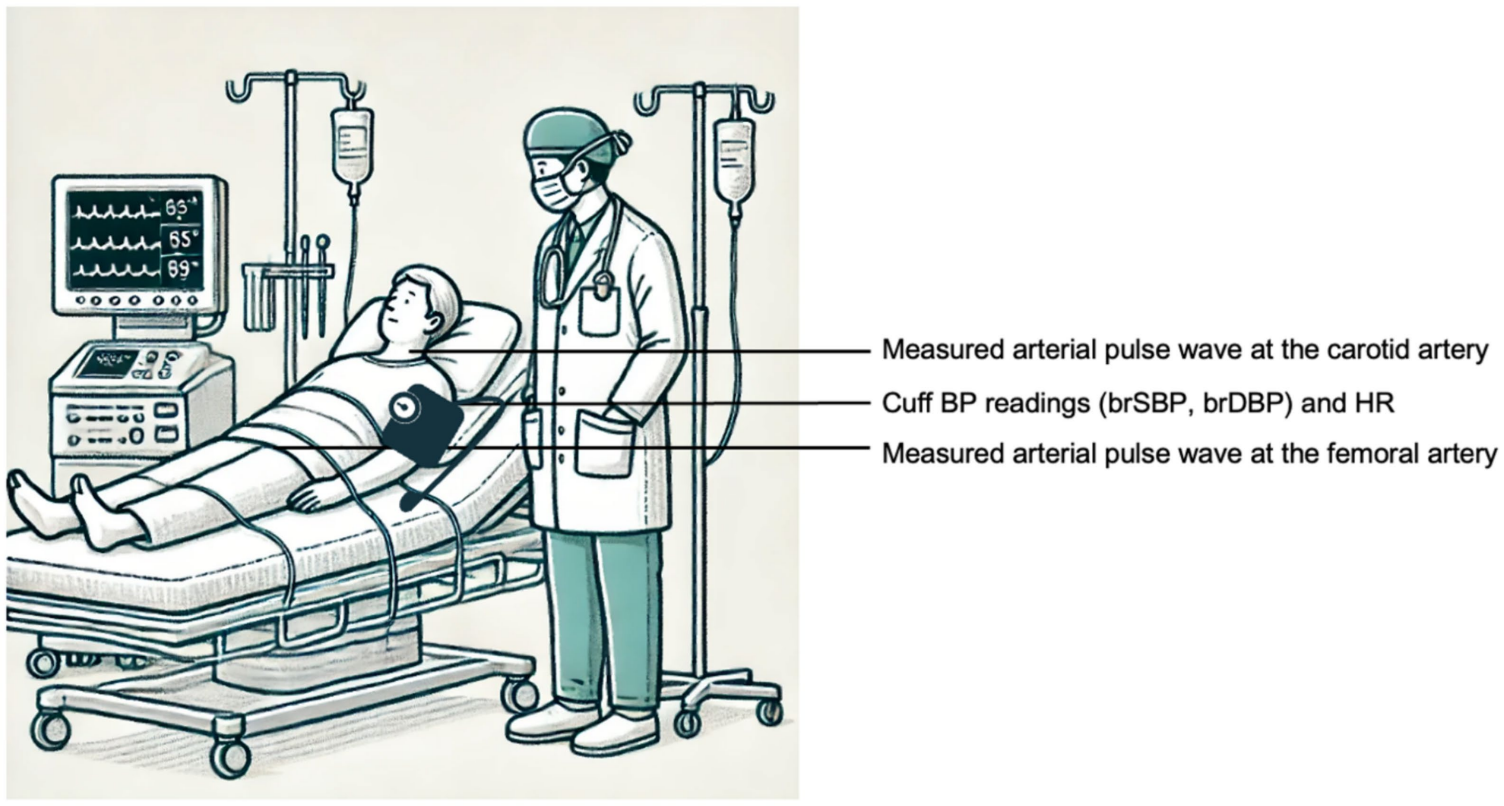
Schematic representation of the measurement setup for the acquisition the input data (brSBP, brDBP, HR, and cfPWV). The animation was created using OpenAI’s GPT-4 model (OpenAI, 2023).

**Figure 7:**
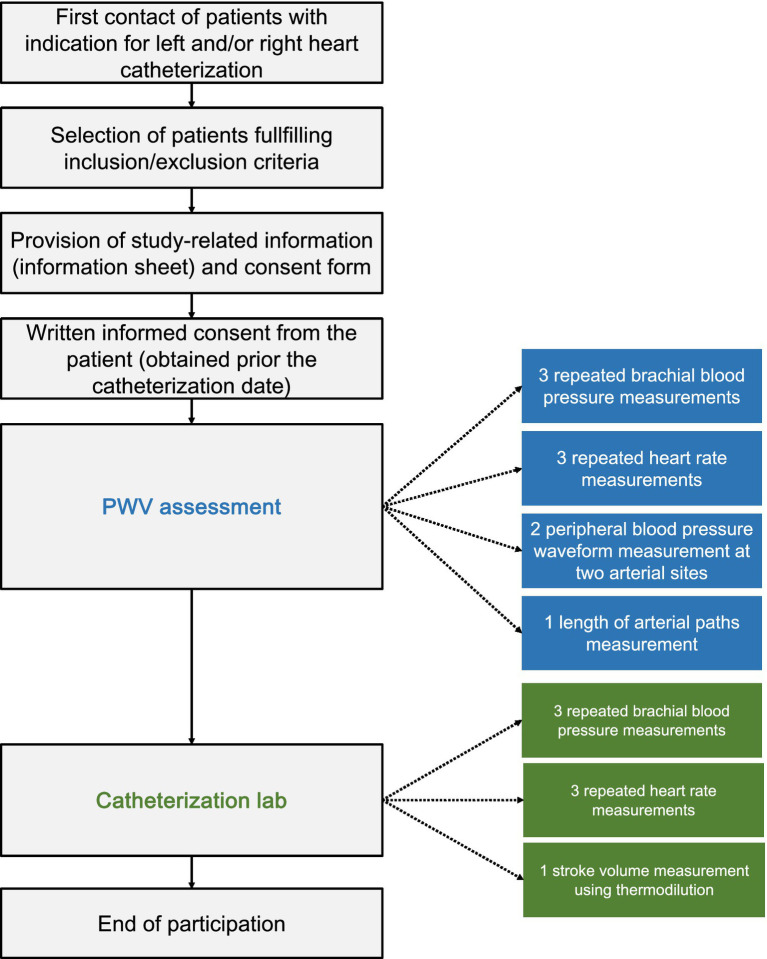
Schematic representation of the study procedures.

Participants that agreed to join the study were initially interviewed to assess their eligibility based on inclusion and exclusion criteria (Supplementary Table S1). Eligible participants received the information sheet and consent form. Before or after heart catheterization, applanation tonometry was performed to acquire the BP waveforms at the right or left carotid, and right femoral arteries to derive the cfPWV. In particular, BP waveforms were acquired non-invasively by the use of two commercially available and validated devices, namely the PulsePen (DiaTecne s.r.l., Milan, Italy)^2^, and the SphygmoCor apparatus (AtCor Medical Pty Ltd., West Ryde, Australia). The intersecting tangent foot-to-foot method was automatically applied to calculate the carotid-to-femoral pulse transit time (PTT) over a minimum of 10 cardiac cycles. The length of the path traveled by the BP waves was estimated approximately as equal to the distance between the arterial sites multiplied by a correction factor of 0.8 (Laurent et al., 2006). As per the guidelines, the measurement was conducted twice. Simultaneously with the applanation tonometry, cuff BP and HR were measured and recorded using a sphygmomanometer of an appropriate cuff size at the right or left brachial artery (three repeated measurements) (Figure 6). In the catheterization laboratory, the thermodilution technique for CO measurement was performed by an experienced professional according to standard procedures. SV was derived as CO/HR. Simultaneously, cuff BP and HR were measured at the right or left brachial artery using conventional sphygmomanometry (three repeated measurements). Data were codified and stored electronically, with uncodified data stored on the hospital’s servers. The study procedures are illustrated in Figure 7.

#### 2.2.3 Data analysis

The collected data was extracted from the designated hospital’s system. For each participant, the three repeated measurements of SBP, DBP, and HR during cfPWV assessment and SV measurement were averaged. The mean value of the two PWV measurements was calculated. We then compared the BP and HR data collected during PWV assessment with those obtained in the catheterization lab to evaluate hemodynamic coherence between the two states. A discrepancy threshold of 25% relative difference was set to assume hemodynamic coherence between the two assessment settings. The threshold was applied for each patient individually for both MAP and HR values. Patients whose MAP and HR profiles differed by more than this threshold between PWV measurement and catheterization were excluded from the analysis. Given the limited size of our patient cohort, we wanted to ensure that we maintain a considerable cohort size after filtering. Hence, the value of 25% was an arbitrary moderate threshold selection. After filtering, the patient data for BP, HR, and PWV were input into our model to predict stroke volume (SV_pred_). To align with the goal of non-invasive clinical assessments using nearly simultaneous PWV and cuff BP measurements, we incorporated BP and HR values recorded during the PWV assessment into the input vector.

### 2.3 Statistical analysis

All data is presented as mean and standard deviation (SD). The statistical analysis was performed in Python (Python Software Foundation, Python Language Reference, version 3.10.9, see text footnote 1). The pairs of BP and HR data obtained during catheterization and PWV assessment were compared using a paired t-test (Student, 1908), while the data from the in silico model was compared to the human cohort using Welch’s t-test (Welch, 1947) to account for the differing sample sizes. The correlation and precision between the estimations (using the linear formula) and the reference data were evaluated using the Pearson’s correlation coefficient (*r*), and the normalized root mean square error (RMSE). The computed normalized RMSE was based on the difference between the minimum and maximum values of the dependent variable (*y*) and was computed as RMSE/(*y*_max_–*y*_min_). Bias and limits of agreement (LoA) (where the 95% of errors are expected to lie) were calculated using the Bland–Altman analysis (Bland and Altman, 1986). Due to the relatively small sample size (<30) and the difficulty in satisfying the normality assumption for the data distribution, we selected the non-parametric Wilcoxon Signed-Rank test (Wilcoxon, 1945), considering a *p*-value <0.05 as the threshold for statistical significance.

## 3 Results

A total of 9,996 hemodynamical profiles were generated, accounting for 833 profile per group (age and gender-based). All profiles were kept to maximize the solution space of the model simulations. Table 3 reports the descriptive characteristics of the generated hemodynamic states.

**Table 3:** Descriptive characteristics of the synthetic cases (*n* = 9,996).

Parameter	Gender	Age groups (833 cases per group)					
		20–29 yrs	30–39 yrs	40–49 yrs	50–59 yrs	60–69 yrs	>70 yrs
Height (cm)	M	175 ± 13	175 ± 13	175 ± 13	175 ± 13	175 ± 13	175 ± 13
	F	176 ± 13	176 ± 13	175 ± 13	174 ± 13	175 ± 13	174 ± 13
Weight (kg)	M	61 ± 6	66 ± 7	65 ± 10	61 ± 5	69 ± 14	61 ± 8
	F	60 ± 6	66 ± 7	65 ± 10	62 ± 5	69 ± 13	62 ± 7
Brachial SBP (mmHg)	M	110 ± 18	114 ± 24	124 ± 24	123 ± 20	139 ± 30	126 ± 27
	F	110 ± 18	115 ± 24	124 ± 24	124 ± 21	142 ± 34	131 ± 29
Brachial DBP (mmHg)	M	65 ± 12	53 ± 14	58 ± 14	63 ± 12	45 ± 15	49 ± 17
	F	65 ± 12	53 ± 14	59 ± 14	63 ± 12	44 ± 16	47 ± 17
Brachial PP (mmHg)	M	45 ± 15	61 ± 30	65 ± 28	60 ± 23	94 ± 40	78 ± 38
	F	45 ± 16	61 ± 30	65 ± 27	61 ± 24	98 ± 45	84 ± 39
MAP (mmHg)	M	80 ± 12	73 ± 11	80 ± 12	83 ± 11	76 ± 9	75 ± 11
	F	80 ± 12	74 ± 11	81 ± 12	83 ± 11	77 ± 10	75 ± 11
Stroke volume (mL)	M	57 ± 18	60 ± 19	55 ± 19	54 ± 17	50 ± 15	45 ± 14
	F	58 ± 18	60 ± 19	56 ± 18	54 ± 17	50 ± 16	46 ± 13
Cardiac output (L/min)	M	4 ± 1.3	3.5 ± 1.1	3.6 ± 1.2	3.6 ± 1.1	3.3 ± 1	3 ± 1
	F	3.9 ± 1.2	3.5 ± 1.1	3.7 ± 1.2	3.6 ± 1.2	3.4 ± 1.1	3 ± 0.9
Heart rate (bpm)	M	70 ± 11	59 ± 7	67 ± 11	68 ± 8	67 ± 7	67 ± 12
	F	69 ± 11	59 ± 7	67 ± 10	67 ± 8	67 ± 7	67 ± 11
Carotid-femoral PWV (m/s)	M	6.4 ± 1.1	8.4 ± 2.3	8 ± 1.9	7.9 ± 1.6	11.6 ± 2.9	11.5 ± 3.6
	F	6.4 ± 1.2	8.4 ± 2.3	7.9 ± 1.8	7.8 ± 1.6	11.5 ± 2.9	11.5 ± 3.4

For the clinical data, out of the 37 patients, 10 were excluded due to missing data (*N* = 8) or erroneous measurements (*N* = 2). Specifically, PWV assessment was not possible for three patients and yielded unrealistic PWV values in two patients, while thermodilution measurement could not be performed in five patients. The three repeated measurements of BP and HR were averaged in all patients, except for one patient, where a single HR measurement was performed during PWV assessment. The subgroup sizes were: $AG_{20-29}^{M}$: 0, $AG_{20-29}^{F}$: 1, $AG_{30-39}^{M}$: 0, $AG_{30-39}^{F}$: 1, $AG_{40-49}^{M}$: 2, $AG_{40-49}^{F}$: 0, $AG_{50-59}^{M}$: 1, $AG_{50-59}^{F}$: 2, $AG_{60-69}^{M}$: 1, $AG_{60-69}^{F}$: 3, $AG_{>70}^{M}$: 8, $AG_{>70}^{F}$: 8. The characteristics of the 27 patients, consisting of 12 (44%) males and 15 (56%) females, are described in Table 4.

**Table 4:** Descriptive characteristics of the patient cohort (*n* = 27^*^).

Parameter	Gender	Age groups					
		20–29 yrs	30–39 yrs	40–49 yrs	50–59 yrs	60–69 yrs	>70 yrs
Height (cm)	M	–	–	180 ± 10	171	183	170 ± 7
	F	165	167	–	166 ± 11	163 ± 10	161 ± 10
Weight (kg)	M	–	–	82 ± 14	78	71	79 ± 11
	F	52	60	–	85 ± 40	48 ± 9	60 ± 8
Brachial SBP_PWV_ (mmHg)	M	–	–	114 ± 16	127	125	138 ± 19
	F	107	107	–	100 ± 4	115 ± 24	125 ± 12
Brachial DBP_PWV_ (mmHg)	M	–	–	80 ± 16	80	68	77 ± 19
	F	58	57	–	61 ± 8	68 ± 16	65 ± 7
Brachial PP_PWV_ (mmHg)	M	–	–	34 ± 1	47	57	61 ± 17
	F	50	50	–	38 ± 12	47 ± 16	60 ± 13
MAP_PWV_ (mmHg)	M	–	–	92 ± 16	96	87	97 ± 17
	F	74	74	–	74 ± 4	84 ± 18	85 ± 7
Brachial SBP_Cath_ (mmHg)	M	–	–	112 ± 19	113	143	145 ± 19
	F	107	102	–	118 ± 26	104 ± 18	132 ± 9
Brachial DBP_Cath_ (mmHg)	M	–	–	78 ± 16	68	85	77 ± 15
	F	65	72	–	70 ± 19	66 ± 6	70 ± 8
Brachial PP_Cath_ (mmHg)	M	–	–	34 ± 4	45	58	68 ± 17
	F	42	30	–	48 ± 6	38 ± 12	62 ± 14
MAP_Cath_ (mmHg)	M	–	–	89 ± 17	83	104	100 ± 14
	F	79	82	–	86 ± 22	79 ± 10	91 ± 5
Stroke volume (mL)	M	–	–	68 ± 7	108	81	82 ± 27
	F	49	124	–	107 ± 7	41 ± 5	64 ± 12
Cardiac output (L/min)	M	–	–	4.6 ± 1.2	7.9	6.1	5.2 ± 1
	F	3.5	10.2	–	8.1 ± 2	3.2 ± 0.6	4.4 ± 0.7
Heart rate_PWV_ (bpm)	M	–	–	68 ± 11	73	75	67 ± 16
	F	71	82	–	76 ± 13	79 ± 10	71 ± 14
Heart rate_Cath_ (bpm)	M	–	–	62 ± 12	74	76	66 ± 17
	F	52	68	–	74 ± 18	80 ± 6	66 ± 14
Carotid-femoral PWV (m/s)	M	–	–	8.1 ± 2.3	5.8	9	12.2 ± 1.7
	F	6.1	5.8	–	7.4 ± 0.4	9.7 ± 1.6	11.6 ± 2

–: Indicates that there are no patients in the group. Single values denote that there is only one patient in the group.

SBP, systolic blood pressure; DBP, diastolic blood pressure; PP, pulse pressure; MAP, mean arterial pressure; X_PWV_, parameter X measured during pulse wave velocity assessment; X_Cath_, parameter X measured during catheterization.

* The table contains the data distributions for all patient with and without considering hemodynamical coherence for BP and HR values between catheterization and PWV assessment. The final analysis was conducted using a subgroup of 24 eligible patients.

For SBP, the brachial SBP in the totality of the model-generated cases was 123 ± 27 mmHg. In the patient data, the SBP measured during PWV assessment (SBP_PWV_) was 124 ± 18 mmHg (Welch’s t-test *p*-value = 0.935), and the SBP measured during catheterization (SBP_Cat_) was 128 ± 21 mmHg (Welch’s t-test *p*-value = 0.284). The brachial DBP in the in silico data was 55 ± 16 mmHg. In the clinical data, the DBP measured during PWV assessment (DBP_PWV_) was 70 ± 14 mmHg (Welch’s t-test *p*-value < 0.0001), and the DBP measured during catheterization (DBP_Cat_) was 73 ± 11 mmHg (Welch’s t-test *p*-value < 0.0001). The mean arterial pressure was 78 ± 12 mmHg for the in silico data and MAP_PWV_/MAP_Cat_ 88 ± 14 mmHg (Welch’s t-test *p*-value < 0.001)/91 ± 13 mmHg (Welch’s t-test *p*-value < 0.0001) for the patient cohort. The heart rate in the model-generated cases was 66 ± 10 bpm. In the patient data, the HR measured during PWV assessment (HR_PWV_) was 71 ± 13 bpm (Welch’s t-test *p*-value = 0.041), and the HR measured during catheterization (HR_Cat_) was 68 ± 14 bpm (Welch’s t-test *p*-value = 0.497). Lastly, SV was calculated to be 54 ± 18 mL for the model-generated cases, while the patient data SV was 74 ± 26 mL (Welch’s t-test *p*-value < 0.001). CfPWV was 9 ± 3 m/s for the model-generated cases and 10 ± 3 m/s for the patient data (Welch’s t-test *p*-value = 0.018).

### 3.1 Comparison of BP and HR values between PWV assessment and catheterization in the patient cohort

Comparing the catheterization (Cat) and PWV measurements, the SBP_Cat_ (128 ± 20 mmHg) was marginally higher than the SBP_PWV_ assessment (124 ± 18 mmHg, paired t-test *p*-value = 0.178). The mean absolute difference SBP_PWV_ and SBP_Cat_ was 12 ± 10 mmHg, with discrepancies ranging from 0 to 39 mmHg. Similarly, the DBP recorded during catheterization (73 ± 11 mmHg) exceeded that of the PWV assessment (70 ± 14 mmHg, paired t-test *p*-value = 0.193). Mean differences for DBP were 9 ± 6 mmHg, with a range from 0 to 24 mmHg. The MAP during catheterization (91 ± 12 mmHg) was also greater than the MAP during the PWV assessment (88 ± 14 mmHg, paired t-test *p*-value = 0.145). For MAP, the mean absolute difference was 9 ± 7 mmHg, with discrepancies between 1 and 24 mmHg. Conversely, the HR_Cat_ (68 ± 13 bpm) was slightly lower compared to the HR_PWV_ assessment (71 ± 12 bpm, paired t-test *p*-value = 0.034), with mean differences of 6 ± 6 bpm and errors in the range of 0–19 bpm. In summary, catheterization typically yielded higher values for SBP, DBP, and MAP, whereas the HR was lower than the corresponding PWV measurements. The scatter and Bland Altman plots comparing the measurements under the two different assessment states are provided in Supplementary Figure S1.

### 3.2 Data analysis and predictions

We selected the SBP, DBP, PP, and MAP values formally used that were obtained during PWV, as those values are the ones that are used in non-invasive clinical assessments. After evaluating the differences in BP and HR, we filtered out cases with high discrepancies between the two settings. Assuming hemodynamic coherence between PWV assessment and catheterization (with an accepted difference of 25%), three patients were considered ineligible for subsequent analysis. Therefore, the analysis was performed for a refined cohort of 24 patients.

### 3.3 Stroke volume estimator

**Figure 8:**
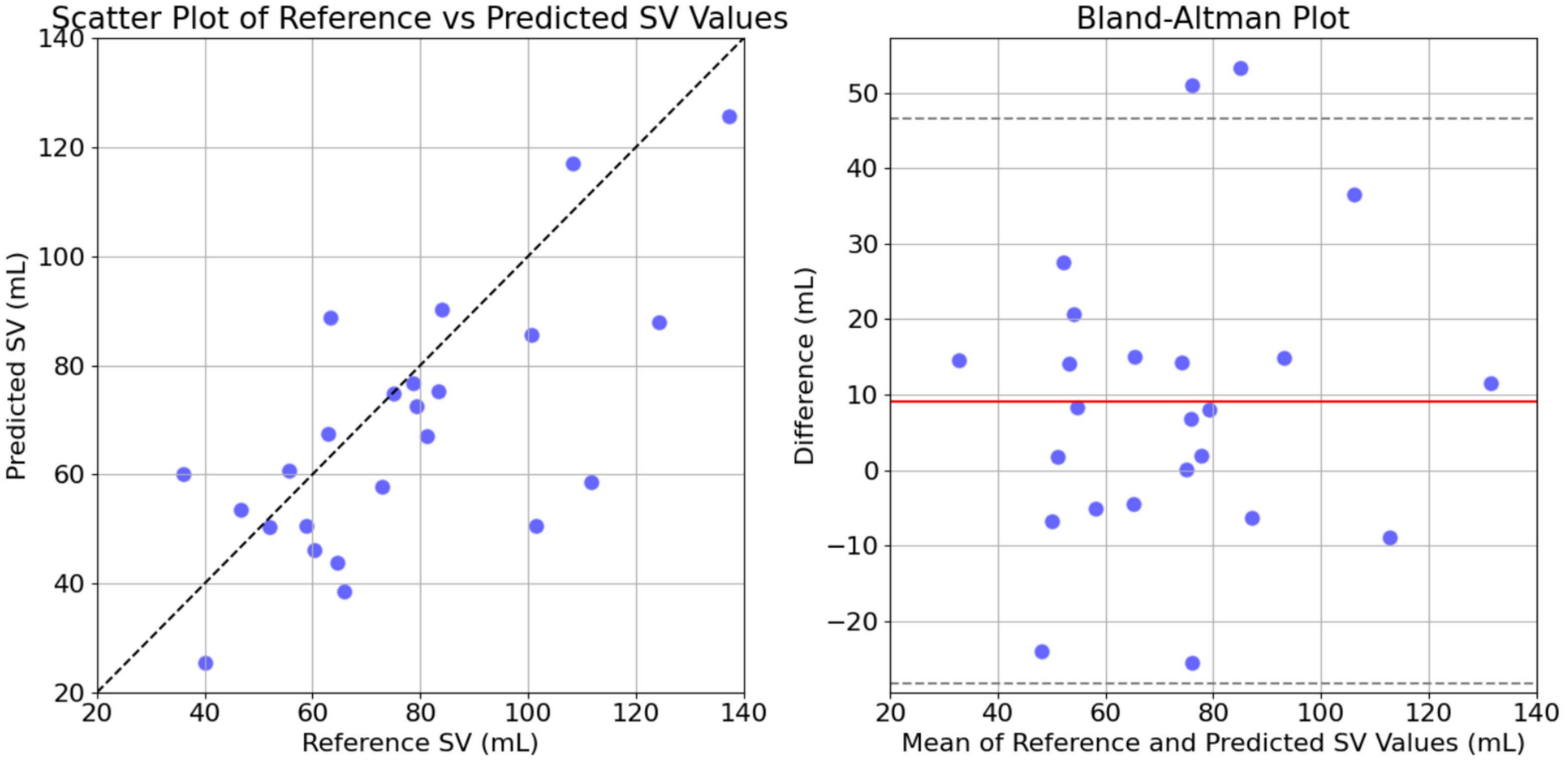
Comparison of the predicted and the reference stroke volume (SV) data for GB model. The left panel shows the scatter plot of predicted SV against reference SV with a line of equality (dashed black line) indicating perfect agreement. The right panel displays the Bland–Altman plot, showing the differences between reference and predicted SV against their mean, with dashed lines for mean difference and limits of agreement (mean ± 1.96 SD).

The GB model achieved satisfactory performance with a correlation coefficient (*r*) of 0.7, an MAE of 16 mL, and an nRMSE of 21%. The bias was −9.2 mL with LoA from −47 to 28 mL. The reference SV was 77 ± 26 mL, and the predicted SV was 68 ± 23 mL, with a *p*-value of 0.025, indicating a significant difference. In this case, the optimal values identified were *‘n_estimators’* (150), *‘learning_rate’* (0.6), *‘max_depth’* (8), *‘max_features’* (sqrt), and *‘min_samples_leaf’* (5). The scatter and Bland–Altman plot are presented in Figure 8.

## 4 Discussion

In this study, we introduced and tested a regression-enabled estimation method for non-invasive stroke volume (SV) estimation. The method demonstrated good agreement with the gold standard invasive thermodilution technique, underscoring its potential for clinical application. Our findings indicate that the proposed machine learning framework, trained on synthetic hemodynamic data, can effectively predict SV using non-invasive measurements. This method enhances our previous inverse problem-solving approach (Bikia et al., 2020; Bikia et al., 2022) by reducing computational costs, making it feasible for real-time clinical applications. The adoption of this method could potentially eliminate the need for complex and costly echocardiographic or MRI assessments.

SV is a primary determinant of cardiovascular function and its assessment, particularly in conjunction with BP measurements, is critical to understand cardiovascular physiology and pathology (Nichols et al., 2011). Continuous SV assessment is essential for diagnosing and guiding therapeutic interventions in patients with heart failure and other critical conditions. These patients often need to undergo multiple invasive procedures and may be exhausted by their disease, treatments, and examinations. In addition, the invasive SV assessment is associated with high cost, specialized equipment and required training of personnel. Hence, it is important to develop precise, reliable, and non-invasive SV monitoring techniques.

While significant progress has been made in the field of BP monitoring, advancements in SV monitoring have been comparatively limited (Jansen et al., 2001; Swamy and Mukkamala, 2008; Fazeli and Hahn, 2012; Ganter et al., 2016; Yazdi et al., 2021). This gap underscores the need to explore new research avenues. Leveraging multiple measurement sources, such as cuff systolic and diastolic brachial blood pressure, and PWV data, can help develop more nuanced and accurate models for SV estimation. These peripheral measurements are non-invasive and can be routinely monitored by any clinician, making them a practical alternative for continuous cardiovascular assessment. Importantly, the latest advances in machine learning for medical applications present an opportunity to revolutionize SV monitoring. By incorporating machine learning techniques, it is possible to create more accurate and accessible models for non-invasive SV estimation. Our study demonstrates the potential of such integration, paving the way for improved cardiovascular monitoring that may enhance patient outcomes by providing reliable, real-time SV assessments without the need for invasive procedures or expensive equipment. Ultimately, such a method could be integrated into a comprehensive apparatus that includes a conventional cuff BP monitor, a tonometric device to acquire PWV measurements, and a user profile analysis. This integration would enable a fast, non-invasive estimation of SV by combining the advanced capabilities of machine learning with routine clinical measurements.

The proposed model demonstrated a moderate correlation with the gold standard invasive thermodilution technique, reflecting its ability to provide reliable estimates of SV using non-invasive measurements in a small human cohort. Despite the promise that these results may hold, the model’s performance leaves room for improvement, particularly in terms of precision and variability. Discrepancies between the estimated SV values and the reference measurements highlight the inherent challenges of using synthetic data and the variability in physiological states, particularly in critically ill patients. To enhance the model’s accuracy, several avenues can be explored. Refining the dataset generation process to include a larger and more diverse patient data (accounting for disease-specific variations in model parameters) would allow the model to learn from a broader range of patho-physiological conditions, improving its generalizability. Refining the machine learning technique, including the exploration of more advanced models, could also help capture the complex relationships between input data and SV, reducing errors. Additionally, incorporating additional non-invasive biomarkers, such as additional measures of arterial stiffness (e.g., carotid-to-radial PWV), would provide a more comprehensive understanding of cardiovascular health and improve the model’s robustness. While the current method demonstrates good potential for clinical application, particularly by eliminating the need for costly and invasive procedures, further development is required to achieve highly accurate, real-time SV estimates across a wider variety of clinical settings.

It should be noted that we observed discrepancies in BP and HR values measured during PWV assessment and catheterization. Our initial hypothesis was that these discrepancies might be minimal, given the controlled clinical environment. However, the results showed that catheterization typically yielded higher SBP, DBP, and MAP values, while HR was slightly lower compared to PWV measurements. These discrepancies stem from differences in the hemodynamical state of the patient under consideration in the different settings of examination. When including the entire dataset (patients exhibiting hemodynamic discrepancies between PWV assessment and catheterization), the prediction model yielded a low correlation of 0.46 (*p* = 0.441), a mean absolute error (MAE) of 21 mL, and wider limits of agreement (−3.2 [−55, 49] mL). This outcome is expected as the model is trained to handle input–output sets that are simultaneous. While simultaneous data is desirable in practical terms, it presents challenges for validation. In particular, validating non-invasive biomarkers against invasive ground truth remains challenging due to the significant variability in the physiological state of critically ill patients.

### 4.1 Limitations

The study has several limitations that need to be acknowledged. This study is limited by the small dataset used for model validation. The data collection was conducted in an invasive clinical setting, which posed several challenges. Recruiting patients for studies involving invasive procedures requires careful coordination of hospital resources, and patient availability. Additionally, the invasive nature of the procedure restricts the number of eligible participants, further limiting the dataset size. These factors, along with the complexity of working with critically ill patients, introduced an inherent difficulty to collect a larger, more diverse cohort. Specifically, critically ill patients often experience exhaustion from continuous monitoring, limiting the ability to subject them to further stress. While these constraints are acknowledged, they highlight the need for larger, multi-center studies to improve the model’s generalizability and accuracy. Future efforts will aim to expand the dataset and address these limitations, enhancing the model’s robustness and applicability across a broader range of patient populations. An important observation is that the patient cohort demonstrates a heterogeneity. This inherent heterogeneity may partly account for the modest correlation observed between invasive measurements and in silico results. Variations in individual characteristics—such as baseline cardiovascular function, age-related changes, and the presence of comorbid conditions—can influence hemodynamic responses and the parameters measured in the clinical setting. This variability may introduce discrepancies when applying a computational model based on averaged physiological assumptions, suggesting that different patient subgroups could exhibit distinct correlations. Future work may benefit from stratifying patients by relevant clinical variables to further elucidate the impact of these factors on model performance.

Moreover, the 1-D model used for in silico hemodynamic profiles represents a healthy individual, while the cohort consists of patients with severe conditions, potentially affecting the mapping relationship between input data and target output. Nonetheless, developing and validating 1-D numerical models specific to different pathologies and clinical conditions, although highly desirable, is challenging. Additionally, the variation of the model parameters relies on normal distributions of physiological data, which might not fully capture the diverse hemodynamic profiles in the patient cohort. However, given that data reported in the literature are typically provided as mean and standard deviation or minimum and maximum values, this approach was reasonable. An additional study limitation is that our model represents the arterial tree, effectively operating as an open model without accounting for the venous circulation. This approach omits a Guytonian perspective of circulation, where the role of venous return is critical in influencing cardiac output and SV. Since SV is significantly affected by venous dynamics, particularly central venous pressure and inferior vena cava measurements, the absence of these parameters in our model may affect the model’s accuracy in SV estimation. Future work will incorporate venous components to provide a more comprehensive representation of the cardiovascular system.

There are also inherent challenges related to data collection, particularly in critically ill patients where measurements are difficult to perform. Achieving concomitant measurements of blood pressure and stroke volume is significantly challenging, and we chose to derive a fair compromise by simulating the simultaneity of the measurements (BP, PWV, and SV) as accurately as possible. Discrepancies in BP and HR values were significant between the PWV and SV assessment settings, yet we addressed them by applying filtering to ensure hemodynamic coherence. Despite these limitations, we believe that both the study’s findings and the dataset provide a unique contribution to the literature. Finally, in future studies, it would be beneficial to assess the method’s accuracy in estimating the relative evolution of SV for a specific patient. This has value in continuous monitoring, where tracking changes over time is often more important than the absolute values (Bikia et al., 2020).

## 5 Conclusion

Our study demonstrated the feasibility of using a machine learning-based approach for non-invasive SV estimation in a small human cohort. The favorable agreement with the invasive gold standard method SV assessment indicates the potential of this technique. However, further research and refinement of the technique are necessary to overcome current limitations and enhance its accuracy and robustness. Integrating machine learning with SV monitoring holds significant potential for advancing patient care by providing a reliable, real-time hemodynamic assessment. This advancement has the potential of improving clinical outcomes by enabling more precise and timely therapeutic interventions, ultimately reducing the need for invasive procedures and enhancing the overall efficiency of healthcare delivery.

## Data Availability

The datasets presented in this article are not readily available because the human data used in the study are subject to privacy regulations and are not publicly available. Requests to access the datasets should be directed to bikia@stanford.edu.

## Footnotes

^1^ http://www.python.org

^2^ www.pulsepen.com
