# Supplementary material for: Testing an inverse modeling approach with gradient boosting regression for stroke volume estimation using patient thermodilution data
Source: Front Artif Intell. 2025 Mar 18;8:1530453. doi: 10.3389/frai.2025.1530453 (PMC11959070; doi:10.3389/frai.2025.1530453)

**Supplemental Materials for**

**Clinical evaluation of an AI-enabled inverse twin method for stroke volume estimation using thermodilution data**

Vasiliki Bikia ^1*^, Dionysios Adamopoulos ^2,3,4^, Georgios Rovas ^1^, Marco Roffi ^2,4^, Stéphane Noble ^2,4^, François Mach ^2,4^, Nikolaos Stergiopulos ^1^

^1^ Laboratory of Hemodynamics and Cardiovascular Technology, Institute of Bioengineering, Swiss Federal Institute of Technology, Lausanne, Switzerland.

^2^ Department of Internal Medicine, Division of Cardiology, Hôpitaux Universitaires de Genève, 1205, Geneva, Switzerland.

^3^ Department of Diagnostics, Division of Nuclear Medicine, Hôpitaux Universitaires de Genève, 1205 Geneva, Switzerland.

^4^ Faculty of Medicine, Department of Medicine, Geneva University, 1206 Geneva, Switzerland.

**Table of Contents**

1. Supplementary Tables
2. Supplementary Figures

## **1. Supplementary Tables**

- **Table S1**: Inclusion and exclusion criteria for study protocol. This table outlines the specific inclusion and exclusion criteria applied to participants in the study, detailing the characteristics required for eligibility as well as those that disqualified potential participants.

| **Inclusion criteria** | |
| --- | --- |
| **1** | Male and female patients aged 18 years old and above, being receiving treatment at the Hospital University of Geneva. |
| **2** | Patients with knowledge of the study language and mental ability to provide an informed consent. |
| **3** | Patients with an indication for left and/or right heart catheterization with measurement of the CO. Typical indications include (list not exhaustive):   1. Investigation of unexplained dyspnea or chest pain. 2. Investigation of patients with heart failure. 3. Investigation of patients with pulmonary hypertension. 4. Investigation of patients with valvular heart disease. 5. Prior to mitral valve repair/replacement. 6. Prior to aortic valve replacement. |
| **Exclusion criteria** | |
| **1** | Patients with cognitive impairment, unable to provide an informed consent. |
| **2** | Patients with documented hemodynamically significant carotid stenosis or documented carotid sinus hypersensibility. |
| **3** | Patients presenting with unexplained syncope. |
| **4** | Patients with significant arrhythmias (e.g., permanent or persistent atrial fibrillation);. |
| **5** | Patients with hemodynamical instability. |
| **6** | Patients with tricuspid regurgitation. |
| **7** | Pregnant women. |

## **2. Supplementary Figures**

- **Figure S1**: Comparison of cardiovascular parameters between PWV and catheterization measurements. Each row represents a different parameter: SBP, DBP, MAP, PP, and HR. The left column shows scatter plots comparing PWV (x-axis) and catheterization (y-axis) measurements, with the green dashed line indicating equality (y = x). The right column displays Bland-Altman plots, illustrating the difference between catheterization and PWV measurements (y-axis) against their mean (x-axis). The red line shows the mean difference, and the gray dashed lines mark the limits of agreement (mean difference±1.96 SD).


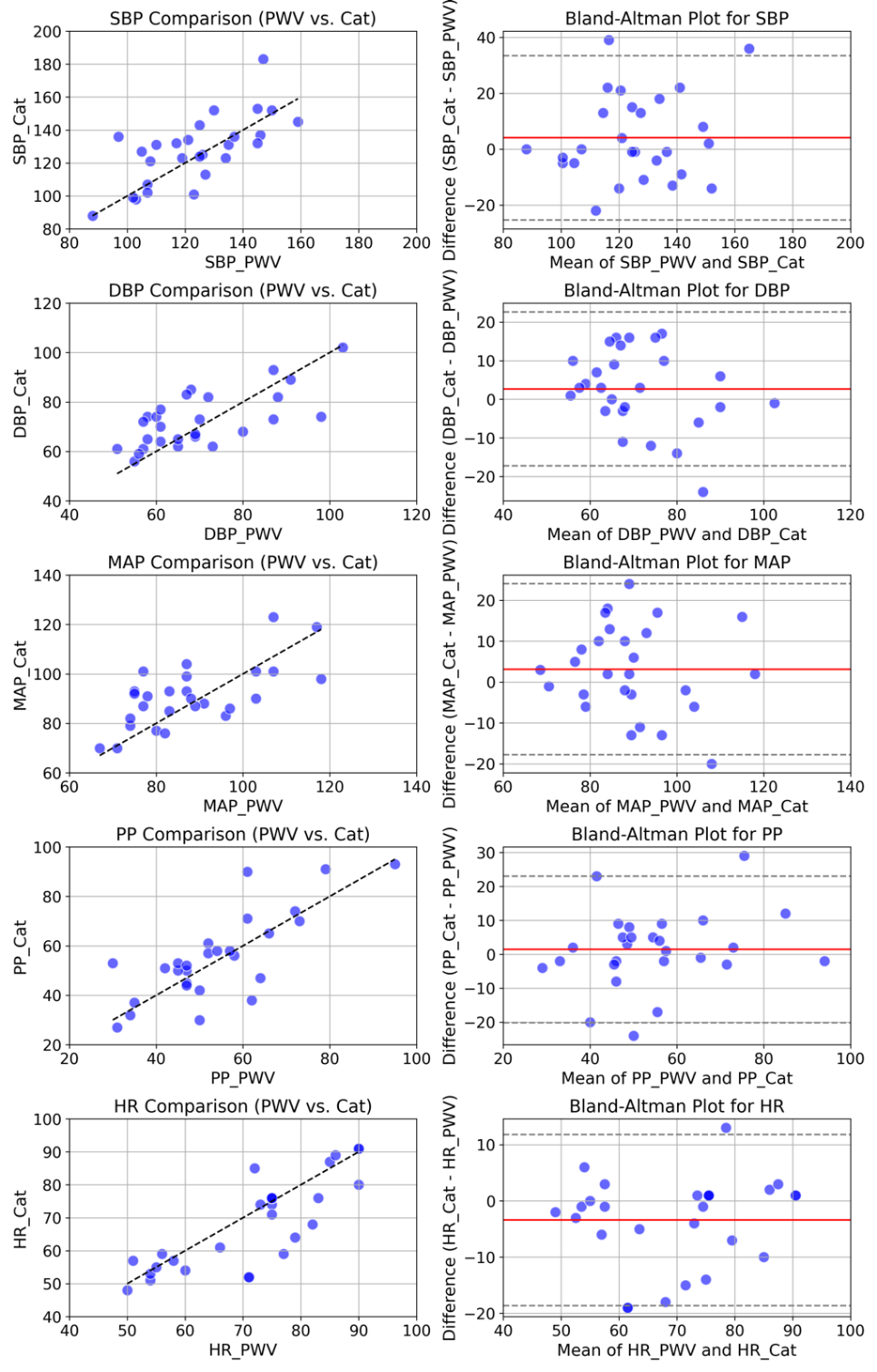

Supplement: Supplementary file 1 [file Data_Sheet_1.docx]
